# Simulating future value in intertemporal choice

**DOI:** 10.1038/srep43119

**Published:** 2017-02-22

**Authors:** Alec Solway, Terry Lohrenz, P. Read Montague

**Affiliations:** 1 Virginia Tech Carilion Research Institute, Roanoke, VA, USA; 2 Department of Physics, Virginia Polytechnic Institute and State University, Blacksburg, VA, USA; 3 Wellcome Trust Centre for Neuroimaging, University College London, London, UK

## Abstract

The laboratory study of how humans and other animals trade-off value and time has a long and storied history, and is the subject of a vast literature. However, despite a long history of study, there is no agreed upon mechanistic explanation of how intertemporal choice preferences arise. Several theorists have recently proposed model-based reinforcement learning as a candidate framework. This framework describes a suite of algorithms by which a model of the environment, in the form of a state transition function and reward function, can be converted on-line into a decision. The state transition function allows the model-based system to make decisions based on projected future states, while the reward function assigns value to each state, together capturing the necessary components for successful intertemporal choice. Empirical work has also pointed to a possible relationship between increased prospection and reduced discounting. In the current paper, we look for direct evidence of a relationship between temporal discounting and model-based control in a large new data set (n = 168). However, testing the relationship under several different modeling formulations revealed no indication that the two quantities are related.

The study of how people trade off value with time has enjoyed a longstanding history spanning several centuries and fields of inquiry, including economics, psychology, and more recently, neuroscience^1^,^2^. Many early theories of intertemporal choice focused on psychological explanations, a trend disrupted by Samuelson’s influential 1937 paper describing the Discounted Utility model^3^, which summarized all of the influences on discounting using a single parameter. Despite early widespread adoption, a large number of problems have been documented with this theory in recent years^4^,^5^,^6^,^7^, shifting the focus to again looking for alternative accounts of behavior. Frederick and colleagues^2^ provide a detailed review of this history.

Despite a long history of study, there is still no agreed upon mechanistic explanation for how such decisions actually arise. A few candidate theories have been proposed towards this end, including the idea that participants optimize reward rate rather than reward magnitude^8^,^9^,^10^,^11^, choose between two different rates of decay^11^ or combine the output of several internal decision making systems with different rates of decay^12^,^13^, estimate the risk associated with waiting, either explicitly or implicitly^9^,^10^,^14^, and several others (e.g. refs 15, 16, 17). However, many potential explanations are complicated by two factors. First, experiments with humans and other animals are often conflated in theoretical description, but it is likely that they index different cognitive mechanisms. Animal experiments usually involve repeated choices between options having the same delays and reward magnitudes, whereas human experiments are ‘one-shot’, posing different, never before experienced options on each trial. Second, theories which can conceivably apply to human data still usually are missing detail. For example, while the idea that estimates of future risk contribute to decision making is attractive, such theories leave open the question of *how* risk estimates are actually computed.

Estimating the future utility of novel choices requires prospection, the ability to project oneself into the future. This ability has recently been studied under the purview of model-based reinforcement learning. Reinforcement learning more generally encompasses a suite of algorithms for optimal learning and decision-making, which over the past two decades have been successful in aiding the study of human and animal behavior, and the neural structures that support it^18^. While most of the focus has been on model-free reinforcement learning, which embodies the common sense notion of ‘habit’, the tide has recently shifted to studying ‘goal-directed’ control or ‘planning’ using the tools of model-based reinforcement learning^19^,^20^,^21^,^22^,^23^,^24^,^25^,^26^,^27^. This framework describes how a learned model, consisting of a state transition function and a reward function, can be integrated on-line to generate decisions. Such a model can be learned directly from experience^21^,^24^,^28^, or can be communicated^29^. In the case of intertemporal choice, the model can be used to project oneself into the future, to estimate where one will be and how they will feel, to better gauge how future reward can be utilized^23^,^30^,^31^. In this way, model-based reinforcement learning provides the necessary ingredients for simulating the long-term utility of never-before experienced options, and has been suggested by several authors as a formal framework for understanding intertemporal choice^23^,^32^,^33^ (see also refs 34 and 35).

Because a model-based controller is able to estimate the likelihood of future states, and as a result can provide a better estimate of future utility, if the brain relies on model-based control to perform intertemporal choice, we may expect to see an inverse relationship between the propensity for performing model-based rollouts and temporal discounting. Individuals that rely more on model-based control should discount the future less. This idea is supported by several empirical studies which show that instructing participants to think about the future during a standard intertemporal choice task decreases the rate of discounting^36^,^37^,^38^,^39^,^40^. Lower discount rates have also been associated with spontaneous task unrelated mind-wandering, which has been argued to be future oriented^41^, and the ability to imagine textual descriptions of future events^42^. Further support comes from work on addiction, which has been associated with decreased goal-directed control^43^,^44^. Of particular note, patients with methamphetamine addiction and binge eating disorder have been shown to exhibit decreased model-based control^45^. At the same time, addiction has been linked to increases in temporal discounting^33^,^46^. Finally, decreased baseline prefrontal dopamine, indexed using a polymorphism in the COMT gene, has been associated with increased discounting^47^ and decreased model-based control^48^.

On the other hand, there is also reason to expect no relationship between model-based control and temporal discounting, or to believe that the relationship may have the inverse direction. With regard to the first possibility, although model-based rollouts allow for better estimates of future utility, this utility may not be greater than that of the (objectively smaller) sooner reward on average. The future can be uncertain or bleak (or one may believe it to be so), and the smaller-sooner reward may carry greater utility for some individuals^34^. With regard to the second possibility, two classes of studies suggest increased model-based control might correspond to increased discounting of the future. First, the administration of L-DOPA has been shown to increase both model-based control^49^ and temporal discounting^50^ (but see refs 51 and 52). Second, disruption of the right dorsolateral prefrontal cortex (DLPFC) using transcranial magnetic stimulation has been shown to impair model-based control^53^, and decrease temporal discounting^54^. Complicating matters further, another study has shown that disruption of right DLPFC induces no change in impulsivity (see also ref. 55 for a third possibility), while disruption of left DLPFC increases discounting^56^. Disruption of left DLPFC has also been shown to decrease model-based control^53^, although there the effect is mediated by individual differences in working memory.

Overall, both theoretical consideration and previous empirical work provide a rather confusing picture concerning the relationship between model-based control and temporal discounting, and although model-based reinforcement learning has been suggested as a candidate mechanism for intertemporal choice^23^,^32^,^33^, there has been no direct empirical work testing this idea. Resolving the status of this relationship has potentially important consequences, especially with regard to the treatment of addiction. There is some evidence that increased temporal discounting and impulsivity more generally may play a causal role in addiction, or that it is at least an antecedent marker^33^,^46^,^57^, but the causal nature of this relationship is ultimately unresolved, partly because it is not clear how intertemporal choice preferences arise. Establishing the causal pathway to addiction is of obvious benefit for developing future therapeutic targets, and understanding the relationship with model-based control represents a potential avenue for progress towards this end.

We sought evidence of a relationship between intertemporal choice preferences and the propensity to deploy model-based control using data from two studies (combined n = 168) in which participants completed two tasks, one designed to measure model-based and model-free control, and another designed to measure temporal discounting. Despite the large size of the data set, and testing the hypothesis under a number of different modeling assumptions, we found no evidence of a relationship between the two quantities.

## Results

The data consist of two studies in which participants completed the same pair of tasks, with minor variations. We first describe the common task structure shared across studies, and then comment on the differences. Each task has been extensively studied and used to measure decision system control^20^,^45^,^53^,^58^,^59^ and intertemporal choice preferences^4^,^7^,^9^,^36^,^37^,^38^,^39^,^40^,^41^,^42^,^50^,^54^,^55^,^56^ in other contexts.

### Two-step task

**Figure 1:**
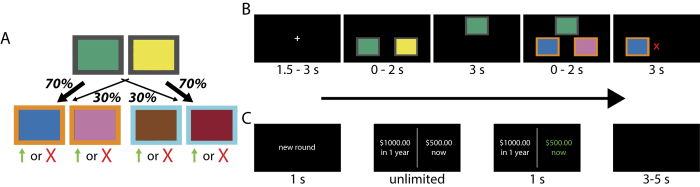
(**A**,**B**) Transition structure and sequence of events within each experimental trial of the two-step task. Each trial involved two decisions. The first-stage decision led probabilistically to a second decision with the probabilities shown in (panel A). At the second stage, each option led to a probabilistic binary payoff whose probability followed an independent Gaussian random walk. The task can be solved using either a model-free (“habitual”) or model-based (“goal-directed”) strategy (see Fig. 2). Images of fractals were used in the actual experiment, here replaced by colored boxes for publication. (**C**) Sequence of events within each trial of the intertemporal choice task. On each trial participants chose between a fixed “later amount” (always $1,000) at varying intervals and a smaller “now” amount of varying magnitudes.

The first task, designed to measure decision system control, is depicted in Fig. 1A,B. On each trial, participants made two binary decisions between fractal images. The first decision was always between the same two images, each image probabilistically leading to one of two other pairs of images. The terminal images resulted in a probabilistic binary payoff. The probabilities governing the transition between stages were fixed throughout the experiment and are depicted in Fig. 1A. In contrast, the probabilities governing the payoffs followed independent Gaussian drifts. Participants had to continually learn which terminal image was best, and plan the best way of getting to it from the first stage.

**Figure 2:**
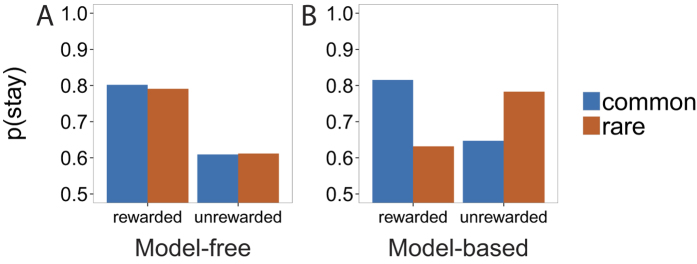
The probability of switching the first-stage decision on two consecutive trials of the two-step task, as a function of whether the previous trial was rewarded, and whether a common or rare transition was experienced between the first and second stage. (**A**) Switch probabilities for a pure model-free agent, which is sensitive only to reward. (**B**) Switch probabilities for a pure model-based agent, which takes the transition structure into account.

The structure of the task, in tandem with computational modeling, allows us to distinguish the contribution of model-free and model-based control to behavior. As a first approximation, intuition regarding our ability to make this distinction can be gained by looking at first-stage choices on consecutive trials, and in particular, whether or not participants stayed with the same choice as a function of the reward and type of transition experienced on the previous trial. Figure 2 plots simulated choice probabilities for a pure model-free and a pure model-based agent. The habitual model-free controller is sensitive only to reward, preferring to repeat rewarded over unrewarded choices regardless of transition type (Fig. 2A). In contrast, the model-based controller learns and maintains a model of the transition structure, allowing for it to be taken into account when making a decision (Fig. 2B).

As an illustrative example, consider what happens when the previous trial was rewarded, but the transition from the first to the second stage was of the rare variety (see Fig. 1). The model-free controller prefers repeating the same action. In contrast, the model-based controller, which has access to the transition structure, knows that in order to maximize the chances of returning to the same rewarding second-stage image, one should switch to the opposite action at the first stage. Given the transition structure, the same first-stage action will only rarely lead to the same second-stage state.

The overall structure of the two-step task was the same in each of the two studies, differing only in terms of a few innocuous parameters: the number of trials per participant (201 in the first study, and 176 in the second study), the rate of the Gaussian payoff drift (0.025 in the first study, and 0.05 in the second study), the reflecting boundaries of the Gaussian drift (0.2 and 0.8 in the first study, and 0.25 and 0.75 in the second study), and the decision deadline (2 s for each stage in the first study, and no deadline in the second study).

### Intertemporal choice task

Participants also completed a standard intertemporal choice task. Each trial featured a binary decision between an amount of money to be received now, and a larger amount of money to be received at a later point in time. As in many previous studies utilizing this task, payoffs were hypothetical^9^,^36^,^37^,^38^,^39^,^54^,^55^. Figure 1C displays the sequence of events in each trial. In the first study, participants completed two separate sessions, with the offers in the second session adjusted separately for each participant according to their indifference point in the first session (for details, see *Methods*). Participants in the second study completed only one session.

The two studies also differed in terms of the duration between the two-step and intertemporal choice tasks (1–674 days in the first study, and the same day in the second study).

### Modeling procedures and results

Different approaches have been used to model data from each of these tasks. In order to ensure that our results were not sensitive to modeling assumptions, we looked for evidence of a relationship under a number of different modeling alternatives. In each case, we used a hierarchical Bayesian modeling procedure to simultaneously estimate per-subject and group level parameters for each task, as well as the relationship between parameters across tasks. Because the relationship we seek evidence for is correlational, we can treat either variable as the “independent” variable and the other variable as the dependent variable. Although the logic of the analysis is similar, each formulation results in a different set of regression parameters with a different posterior geometry. In practice, we found that treating the quantities from the two-step task as the “dependent” variables allows for more robust sampling of the posterior, and this is the approach we adopted in the analysis below. Performing the analysis the other way yields similar results.

We modeled the data from the two-step task using both a logistic regression model looking at pairs of choices on consecutive trials (as described above, see also refs 20, 45, 53, 58 and 59), and using a hybrid reinforcement learning model that takes the participants’ full decision history into account^20^,^45^,^58^. Although it is now widely agreed that hyperbolic discounting is a more appropriate description of intertemporal choice data than exponential discounting^4^,^9^,^38^,^40^,^42^,^50^,^54^,^60^, we modeled this data using both functions. Finally, some studies have suggested that discount rates in the intertemporal choice task are right-skewed at the group level^9^,^38^,^40^,^60^. It is not clear whether this is effect is real, or an artifact of older model fitting procedures that fit the data from each subject separately. To account for both possibilities, we crossed each model variation described above with using both a log-normal and a normal distribution to model group level discount rates. In all, this resulted in eight models to test (2 two-step models × 2 discount functions × 2 discount rate group distributions). Modeling details are provided in *Methods*.

An orthogonal dimension with potential influence on our results concerns how the data are aggregated. To reduce posterior variance and maximize power, given the similarity of the tasks across the two studies, it seems sensible to combine all of the data together and analyze it all at once. However, it is also possible that the small differences between studies could interact in unforeseen ways, and lumping the data together could hide effects that are present in the individual studies. To account for this possibility, we fit each of the eight models to three different partitions of the data, first combining all of the data together, and then separately analyzing each of the two studies.

**Figure 3:**
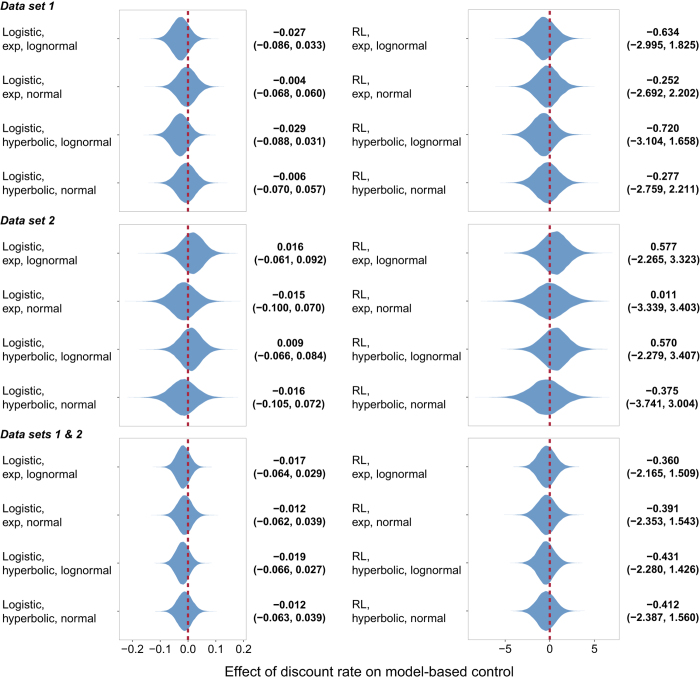
Violin plot of the posterior distribution of the regression coefficient modeling the effect of discount rate in the intertemporal choice task on model-based control in the two-step task under each model formulation. Beside each plot is the median value and the 95% credible interval.

**Figure 4:**
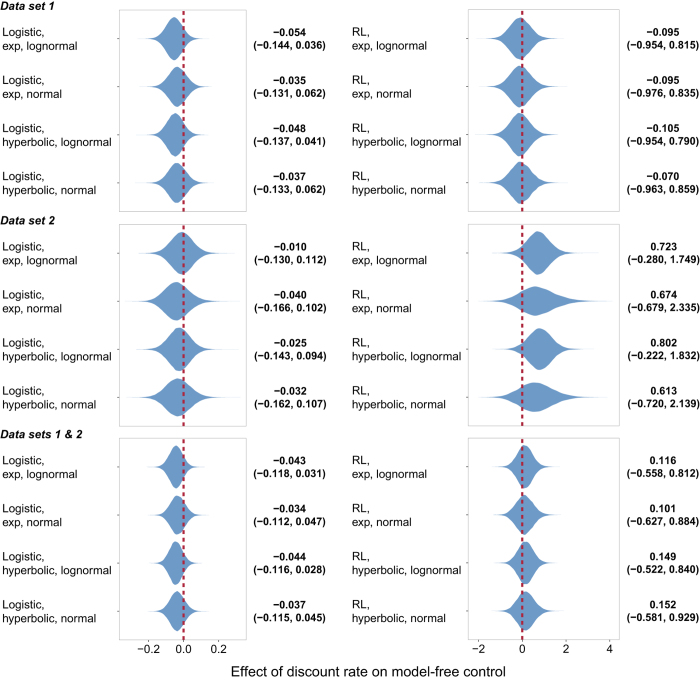
Violin plot of the posterior distribution of the regression coefficient modeling the effect of discount rate in the intertemporal choice task on model-free control in the two-step task under each model formulation. Beside each plot is the median value and the 95% credible interval.

**Figure 5:**
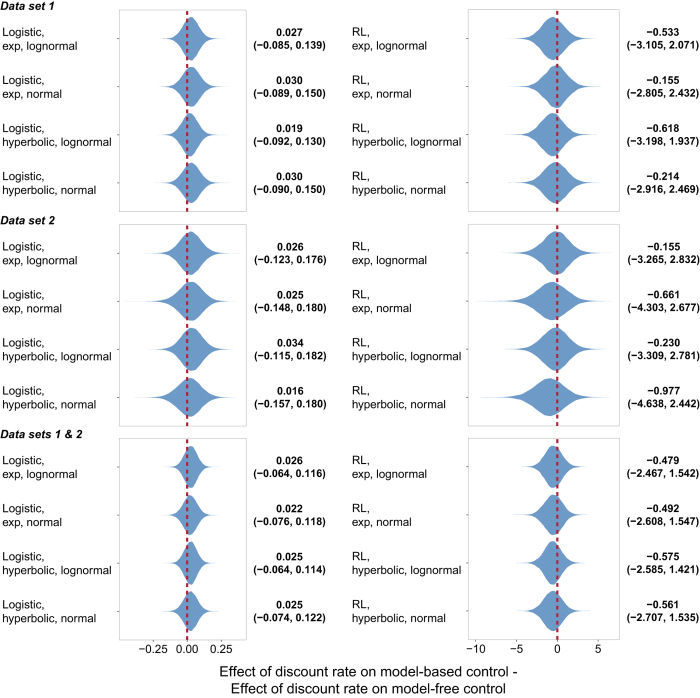
Violin plot of the posterior distribution of the differential effect of discount rate in the intertemporal choice task on model-based control in the two-step task under each model formulation. Beside each plot is the median value and the 95% credible interval.

**Figure 6:**
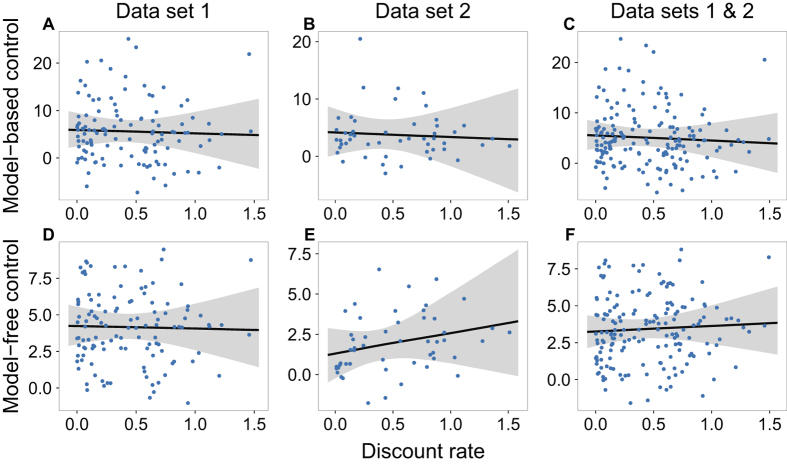
The relationship between discount rate in the intertemporal choice task and the propensity to deploy each decision system in the two-step task using a reinforcement learning model for the two-step data, a hyperbolic discount function, and a normal group level distribution for discount rate. Each dot represents the median of the respective parameter estimates. The black line is the median regression line, and the gray area outlines its 95% credible interval. To understand the scale of the discount rate, note that delay in the models was scaled to the maximum in the experimental data (one year).

In both studies, participants on average used both decision systems to solve the two-step task (Supplementary Figs 1 and 2). However, none of the 24 analyses (8 models × 3 partitions of the data) yielded any evidence of a relationship between either model-based control or model-free control and temporal discounting. Figures 3 and 4 display the estimated effect of discount rate on model-based and model-free control, respectively, under each formulation. In each case, there is substantial posterior mass near 0. Figure 5 plots the difference between the effect on model-based and model-free control, an estimate of the specific influence of discount rate on model-based control. Here too there is substantial posterior mass near 0 under each formulation. Finally, Fig. 6 provides a close-up view of these results for a representative analysis (reinforcement learning model of the two-step data, hyperbolic discount function, and normal discount rate distribution), showing scatter plots of the relationship between discount rate and each decision system under each partition of the data.

We also reran all 24 analyses after first fitting the reinforcement learning model separately to data from each study, and including only participants whose 95% credible interval for model-based control, model-free control, or control at the second stage (*β*_*mb*_, *β*_*mf*_, and *β*_2_, see *Methods*) included 0. Such a scheme approximates removing participants who did not engage with the two-step task at either decision stage. This left 105/117 participants in Study 1 and 42/51 participants in Study 2. Restricting the analysis in this way did not reveal any hidden evidence of an effect on model-based control (Supplementary Figs 3–5). Two of the eight models suggested an effect on *model*-*free* control in Study 2. However, this result did not replicate in Study 1, or when looking at the data from both studies together.

## Discussion

Previous theoretical work has promoted model-based reinforcement learning as a candidate framework for describing how people make one-shot decisions involving the future^23^,^32^,^33^. A number of empirical studies have provided support for this idea, including studies manipulating future orientation^36^,^37^,^38^,^39^,^40^ (‘episodic future thinking’), studies testing the relationship between temporal discounting and mind wandering^41^, and imagination^42^, studies testing the relationship between model-based control, temporal discounting, and a polymorphism in the COMT gene^47^,^48^, and finally, studies testing how model-based control and temporal discounting differ in patients with addiction^43^,^44^,^45^,^46^. Taken together, this work has suggested an intuitive hypothesis: People that are more model-based (i.e. those that engage more in prospective planning) should discount the future less. A possible mechanism mediating this relationship is the model-based controller’s ability to predict candidate future states, allowing the decision maker to simulate where they will be and how they will feel^23^,^30^,^31^, and as a result, better evaluate what a future offer is really worth.

On the other hand, studies on the effects of L-DOPA^49^,^50^ and stimulation of prefrontal cortex^53^,^54^ have suggested the opposite relationship. Complicating matters further, there may be a normative reason for an agent apt at simulating the future to still prefer smaller-sooner rewards^34^, if the simulations reveal a bleak or uncertain future where later reward may not materialize. To elucidate the nature of the relationship between model-based control and temporal discounting, we analyzed data from two studies in which participants completed two standard tasks designed to measure each variable of interest. We looked for evidence of a relationship using a number of different modeling assumptions and dividing the data in a number of different ways. However, we find no evidence of a relationship. There was also no evidence of a relationship between model-free control and temporal discounting.

Although these results appear inconsistent with studies of episodic future thinking in intertemporal choice, it should be noted that such studies show that discounting is attenuated when participants are explicitly prompted to think about the future, compared to when they are left to make decisions on their own accord. The current work does not manipulate decision context in this way directly, and instead tests whether spontaneous orientation towards the future indexes discounting. It is not clear why explicit and spontaneous orientation should affect temporal discounting in different ways. However, one possibility is that intertemporal choices are made using a mechanism other than prospection (see below) at baseline, and explicit future orientation can additively influence choice.

The results are also at odds with two other studies that do address spontaneous prospection, albeit indirectly. Smallwood and colleagues^41^ looked at the relationship between temporal discounting and mind-wandering, which they argue to be future oriented, and Lebreton and colleagues^42^ analyzed the relationship between temporal discounting and individuals’ self-reported ability to imagine textual descriptions of hypothetical future outcomes. Both studies suggest that enhanced orientation towards the future correlates with less temporal discounting.

Also unforgiving are studies which show that individuals with addiction are both less model-based and tend to discount the future more^43^,^44^,^45^,^46^, that reduced prefrontal dopamine indexed using a polymorphism in the COMT gene correlates with reduced model-based control and increased temporal discounting^47^,^48^, and that (in contrast) the administration of L-DOPA^49^,^50^ and the stimulation of prefrontal cortex^53^,^54^ drive model-based control and temporal discounting in the same direction. Although the first two sets of studies are themselves at odds with the second set, all of them are at odds with the results seen here, where we find no evidence of any relationship between the two quantities. At present, it is not clear how to reconcile these findings.

A potential concern is that, despite the size of our combined data set, the analysis is simply not powerful enough to detect evidence of a relationship. Perhaps especially suspect is the fact that in the first study, participants performed the two tasks on different days. Two types of evidence speak against these concerns. First, although the stability of model-based control as measured using the two-step task has not been previously investigated, the stability of temporal discounting has been tested, and it has been shown to be stable even after a year^61^,^62^,^63^. In general it is considered to be a persistent trait variable^61^,^64^. Second, restricting our analysis to data from the second study, in which participants completed both tasks on the same day, also revealed no evidence of a relationship. This is despite the fact that the size of the second study alone (n = 51), although smaller than the first, is on par with other studies utilizing the two-step task^20^,^58^,^59^,^65^,^66^,^67^ and the temporal discounting task^7^,^68^,^69^,^70^,^71^,^72^ (although it should be noted that some of the latter studies are on individuals with addiction).

From first principles, it is not clear that being able to simulate the future should necessarily bias choice one way or the other^34^. On one hand, if both the present and future are relatively bright, meaning that the smaller-sooner reward is not critical for survival, and that the larger-later reward is likely to materialize, then it makes sense to prefer the later reward. On the other hand, if either the present or the future is bleak, one should take what they can now. If likely future outcomes are not biased in either direction in the population, we should not expect to observe any relationship between model-based control and temporal discounting on average. It is also possible that the measure of model-based control indexed by the two-step task does not translate into the type of prospection used during intertemporal choice, and a unified task simultaneously indexing both quantities has to be designed. However, not only is the latter account unappealing on grounds of parsimony, neither explanation would solve the mystery of why previous work treating each quantity in isolation has suggested a relationship.

Very different, alternative, mechanistic explanations for intertemporal choice include decision by sampling^73^,^74^ and the use of simple heuristics^75^,^76^. Decision by sampling attempts to explain the shape of a variety of neuroeconomic utility functions using a single simple mechanism based on binary comparisons between the item in question and similar items in memory. Empirically, the theory has begun to be applied to the study of loss aversion^74^, but has not yet been tested in the context of intertemporal choice.

The idea that intertemporal choice is based on simple heuristic preferences rather than explicit simulations of the future is at least 15 years old^76^, and has recently been reinvigorated^75^. In short, in the latter scheme decisions are made based on a feature vector consisting of simple functions (addition, division) of the two options along each dimension (money and time). The features combine linearly to yield a probabilistic preference. Other heuristics may be simpler still. For example, Stevens^11^ has proposed that some animals may have evolved to cache food in response to environmentally driven biological factors. Such actions are future oriented, but do not require actually forming explicit representations of the future.

It should be noted that neither the simulation theory nor either of the two alternatives described above, if true, would fully describe the entirety of the variance in intertemporal choice. Other cognitive variables and individual differences are likely to be in play, including differences in memory retrieval, attention, and visceral processing^77^.

The current work, along with the possible alternative mechanistic explanations described above, raises the broader question of whether the intertemporal choice paradigm is an appropriate proxy for studying real life trade-offs between value and time. The answer could very well be yes, and that real life trade-offs rely on mechanisms that do not require explicitly constructing a representation of the future, or that building such a representation can have mixed effects. However, we should also be open to the possibility that there are important additional aspects of real life behavior that the intertemporal choice paradigm does not capture, in which case effort should be extended to designing complementary experimental paradigms.

We cannot answer this question in the current paper, but end by highlighting a few obvious ways in which real life decisions differ from the laboratory study of intertemporal choice. As a motivating example, consider the decision of whether to take a job out of college or to pursue a Master’s degree. First, there is ambiguity in value. One can estimate the value of each option based on published average statistics, opportunities for jobs now vs. later through personal networks, and so on, but it is difficult to exactly quantify the payoffs and uncertainty associated with the decision. Second, outcomes are probabilistic and there is time-independent risk in addition to time-dependent risk. One could end up hating their first job out of college, or the company could fold. Further, the probabilities themselves are also ambiguous. Third, in life there is often opportunity to hedge risk, especially time-dependent risk. If there is a potential job on the line, one can go through the interview process to obtain a favorable recommendation, and then elect to pursue a Master’s degree anyway. If the degree doesn’t work out, it may be possible to come back to the ‘smaller-sooner’ reward. Fourth, real life decisions are often abstract, living at a high level of hierarchy. And finally, important real life decisions involve even longer time scales than the ones studied in the laboratory. Withholding $200 now to get $500 in two years may buy more things, but a Master’s degree could pay dividends for a lifetime. The benefits may be both direct, but also indirect, allowing for opportunities that may not otherwise be present. More formally, they may allow one to visit parts of the state space they may not otherwise be able, a decision for which simulation seems especially important.

That the intertemporal choice task does not have these properties may be a feature, in that it simplifies the problem while still invoking the same mechanisms driving choice. Or it could be a bug, instead relying on an orthogonal set, or a small subset, of the relevant mechanisms.

## Methods

### Participants

One hundred and seventeen (117) participants completed the first study and fifty-one (51) participants completed the second study, each a component of the Roanoke Brain Study, a large scale data collection effort to study individual differences. All experimental procedures were approved by the Institutional Review Board at Virginia Tech, and experiments were carried out in accordance with the approved guidelines and the regulations set forth by this board, including obtaining informed consent. All participants were included in the analysis.

### Two-step task

Our version of the two-step task is similar to the one used by other groups^20^,^45^,^53^,^58^,^59^. On each trial, participants made two binary decisions, with each option represented by a fractal image. The outcome of the first-stage decision probabilistically led to one of two second-stage decision states, as shown in Fig. 1A. The second-stage decision resulted in a probabilistic binary payoff (1 or 0) whose probability followed an independent Gaussian random walk for each terminal option (fractal image). The random walk had reflecting boundaries at 0.2 and 0.8 in the first study, and 0.25 and 0.75 in the second study, and a standard deviation of 0.025 in the first study, and 0.05 in the second study. Participants were awarded an extra $0.10 per point earned. The sequence of events within each experimental trial is shown in Fig. 1B. In the first study, each stage of decision had a two second deadline, resulting in the trial being aborted if either was missed. The second study had no deadline at either stage. The studies also differed in the number of trials each participant completed, which numbered 201 in the first study and 176 in the second study.

### Intertemporal choice task

We used an adaptive intertemporal choice paradigm^78^, with the sequence of events during each trial shown in Fig. 1C. Participants in the first study completed two sessions, and those in the second study completed one. The first session was the same in both studies. On each trial participants made a binary decision between receiving an amount of money now, and a larger amount later. As in numerous previous studies, payoffs were hypothetical^9^,^36^,^37^,^38^,^39^,^54^,^55^. The ‘later’ amount was fixed to be $1,000. Trials were blocked by delay, with delays of 1 day, 1 week, 1 month, 3 months, 6 months, and 1 year. Block order was selected pseudo-randomly. There were 6 trials for each delay, with a staircase procedure used to adjust the ‘now’ amount. The first ‘now’ offer was always half the later amount (i.e. $500), and $250 was either added or subtracted from this amount on the next trial depending on whether participants selected the ‘later’ or ‘now’ option, respectively. That is, if participants selected ‘later’ on the first trial, the second trial was a choice between $750 now and $1,000 later, and similarly, if participants selected ‘now’ on the first trial, the second trial was a choice between $250 now and $1,000 later. Increments (or decrements) on subsequent trials were half the increment (or decrement) on the previous trial (e.g. the increment or decrement following the second trial was $125, following the third trial it was $62.50, and so on). This procedure resulted in a continual refinement of the discount estimate.

In the first study, participants completed a second session of the intertemporal choice task on the same day to further refine their discount estimate. The general procedures were identical to the first session, except for how the ‘now’ amount was selected and how the trials were blocked. For each delay, the ‘now’ amounts were selected relative to the ‘now’ amount following the increment or decrement on the last trial of the first session. Three trials were made to have ‘now’ amounts above this amount, and three below. The amounts were spaced using bins defined as follows. If the reference ‘now’ amount was not near the floor ($0) or ceiling ($1000), the bin width was defined to be $10. If it was more than $970 (3 times the default $10 bin width), the bin width for amounts above was


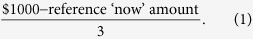


If it was less than $30, the bin width for amounts below was


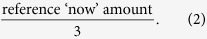


The first ‘now’ amount above was then


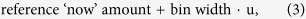


where *u* is a uniform random number between 0 and 1. The second ‘now’ amount above was


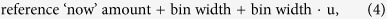


and the third ‘now’ amount above was


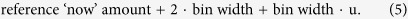


The amounts below were similarly defined. Trials were no longer blocked by delay and were instead shuffled pseudo-randomly across delays.

### Two-step regression analysis

The logistic regression and reinforcement learning model analysis of the two-step task follows previous work^20^,^45^,^53^,^58^,^59^.

The logistic regression took the following form:


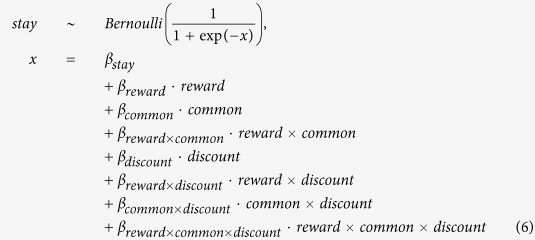


The variable *stay* took on value 1 or 0 depending on whether or not the same first-stage action (fractal image) was chosen on the previous trial. *reward* took on value 1 or −1 depending on whether the previous trial was rewarded, and *common* took on value 1 or −1 depending on whether the transition between the first and second stage on the last trial was common or rare. *discount* is the z-scored discount rate for each participant, estimated using one of the models described below. The term reflecting the interaction between reward and discount rate represents the extent to which discount rate affects stay behavior *in the direction predicted by the model*-*free system*, and similarly for the three-way interaction of reward, transition, and discount for the model-based system.

The regression was performed using a hierarchical Bayesian formulation. *β*_*stay*_, *β*_*reward*_, *β*_*common*_, and *β*_*reward*×*common*_ were instantiated once per participant, each drawn from a group level Gaussian with a broad *N* (0, 2^2^) prior on the mean and a half-Cauchy(0, 2.5) prior on the standard deviation. The remaining regression coefficients were instantiated once at the group level with a *N* (0, 2^2^) prior. The group level estimate of *β*_*reward*_ is a measure of model-free control, as a model-free agent is sensitive to reward regardless of transition type (see main text). Likewise, the group level estimate of *β*_*reward*×*common*_ is a measure of model-based control. The interaction of each term with the discount rate represents how much it influences each system, above and beyond their main effects on choice behavior.

### Two-step reinforcement learning model

The model-free component learned a table of action values, *Q*(*s, a*). The environment consisted of three primary states, one for the first-stage decision, and one for each possible second-stage decision, and two actions in each state, corresponding to the fractal images. Q-values were initialized to 0.5 (mid-way between the two known extreme values) and updated according to SARSA(*λ*)^79^:


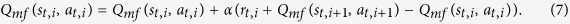


*t* refers to the trial number and *i* to the decision stage. *r*_*t*,*i*_ is the immediate reward, always 0 following the first stage, and 1 or 0 following the second stage. *Q*_*mf*_(*s*_*t*,3_, *a*_*t*,3_) was set to 0 because there was no third stage. An eligibility trace updated first-stage Q-values according to the second-stage outcome:


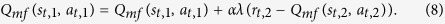


Traces were reset at the beginning of each trial. For simplicity, given the short two-step duration of each episode, we set *λ* to 1 rather than fitting it as a free parameter.

Non-chosen action values decayed to baseline:


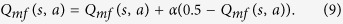


At the second stage, the model-based controller used the same temporal-difference learning rule, and *Q*_*mb*_(*s*_*t*,2_, *a*_*t*,2_) = *Q*_*mf*_(*s*_*t*,2_, *a*_*t*,2_). Following previous work, the transition function used the veridical values (0.7 and 0.3), and the mapping of the first-stage action to the predominant second-stage state was assigned based on the difference between the number of times the first action led to the first second-stage pair plus the second action led to the second second-stage pair, and the number of times the opposite transitions were observed. A single backup operation using the Bellman equation was used to combine the reward and transition functions and compute model-based action values at the first stage:


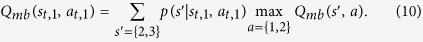


Action selection was conducted using a softmax choice rule. At stage one:


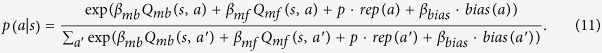


The function *rep*(*a*) is 1 when *a* is the action taken during the first stage of the previous trial, and 0 otherwise. *p* captures the tendency to repeat (*p* > 0) or switch (*p* > 0) actions irrespective of value. The function *bias*(*a*) is 1 for the second action (arbitrarily chosen) and 0 for the first action. This incorporates bias towards the first action when *β*_*bias*_ is negative.

At the second stage, action selection was dependent on a single set of Q-values:


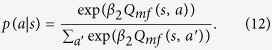


There were six parameters in all, *α, β*_*mb*_, *β*_*mf*_, *β*_2_, *p*, and *β*_*bias*_. Each parameter was instantiated separately for each participant. Subject level parameters were modeled as being drawn from a group level Gaussian similar to the regression model above. An exception to this are the bias parameters, which captured individual nuance and had independent Gaussian priors. Parameters governing the strength of model-based and model-free control also incorporated the effect of discount rate:


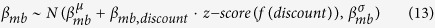


and similarly for *β*_*mf*_. The learning rate, *α*, was transformed to the (0, 1) range using the logistic function before being applied. The function *f*(·) was the identity function when the group level discount distribution was normal, and it was the log function when the group level discount distribution was log-normal (see below). The hyperprior on each group level mean was a broad *N*(0, 10^2^) Gaussian (with the exception of the group learning rate, which had a *N*(0, 5^2^) prior), with a half-Cauchy(0, 2.5) for the standard deviation.

### Intertemporal choice task models

We tested two different discount functions in modeling the intertemporal choice data, exponential discounting:


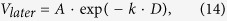


and hyperbolic discounting:


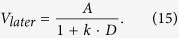


Here *A* is the veridical amount of the offer, *D* is the delay scaled to the maximum available (one year), and *k* is the discount rate. Action selection was conducted according to a softmax choice rule:


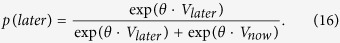


Each model had two parameters, *k* and *θ*, instantiated once for each participant. At the group level, the discount rate *k* was separately modeled in two different ways for each discount function, one as a half-Gaussian defined on [0, ∞) and the other log-normal. The parameter *θ* was modeled as a half-Gaussian defined on [0, ∞). Broad hyperpriors for the group means were defined relative to the scale of each parameter, *N*(0, 2^2^) for the discount rate, and *N*(0, 10^2^) for *θ*. The hyperprior for the group level standard deviation parameters was half-Cauchy(0, 2.5).

### Model fitting

We fit eight different models, crossing each way of modeling the two-step task (logistic regression, reinforcement learning model) with two discount functions (exponential, hyperbolic) for the intertemporal choice data, and two different assumptions about the group level distribution of discount rates (normal, lognormal). We separately fit each model to the combined data from both experiments, and to the data from each experiment.

Inference for each model was performed via Markov chain Monte Carlo, using the No-U-Turn sampler^80^ implemented in Stan (Stan Development Team). Proper mixing was assessed by ensuring the 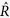 statistic was less than 1.1 for all variables^81^,^82^, and qualitatively by eye. Eight chains were run in parallel for 4,000 samples (10,000 for the regression models), using the first 1,000 for warmup. The posterior was estimated with the resulting 24,000 samples (72,000 for the regression models).

## Additional Information

**How to cite this article:** Solway, A. *et al*. Simulating future value in intertemporal choice. *Sci. Rep.* **7**, 43119; doi: 10.1038/srep43119 (2017).

**Publisher's note:** Springer Nature remains neutral with regard to jurisdictional claims in published maps and institutional affiliations.

## Supplementary Material

Supplementary Figures
