# Supplementary material for: Simulating future value in intertemporal choice
Source: Sci Rep. 2017 Feb 22;7:43119. doi: 10.1038/srep43119 (PMC5320483; doi:10.1038/srep43119)
Supplement: Supplementary Figures [file srep43119-s1.pdf]

# Simulating future value in intertemporal choice

Alec Solway[1], Terry Lohrenz[1], and P. Read Montague[1,2,3]

[1]Virginia Tech Carilion Research Institute, Roanoke, VA, USA
[2]Department of Physics, Virginia Polytechnic Institute and State University, Blacksburg, VA, USA
[3]Wellcome Trust Centre for Neuroimaging, University College London, London, UK

# Supplementary Figures

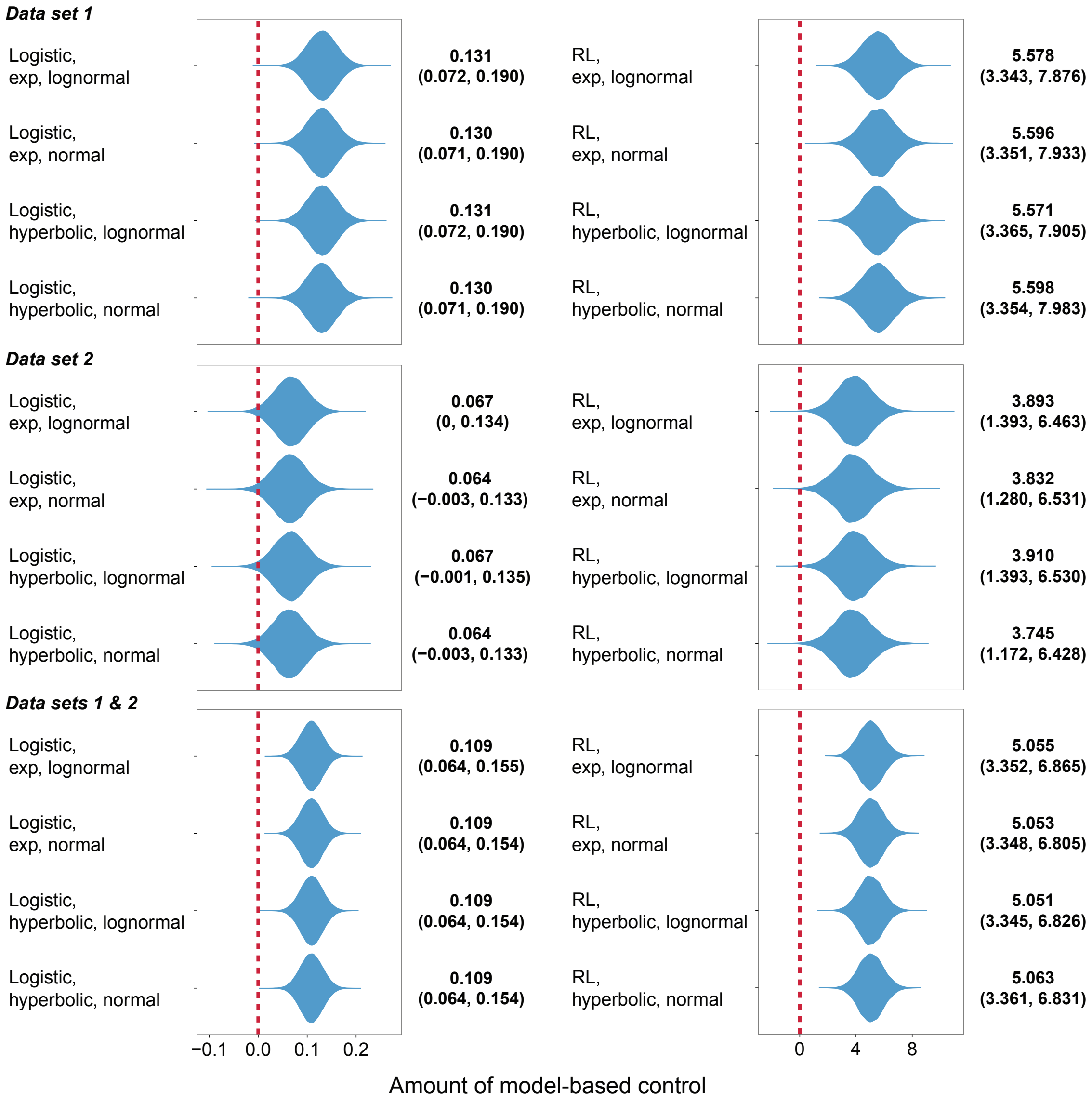


Figure S1: Violin plot of the posterior distribution of the estimated amount of model-based control in the two-step task under each model formulation. Beside each plot is the median value and the 95% credible interval.

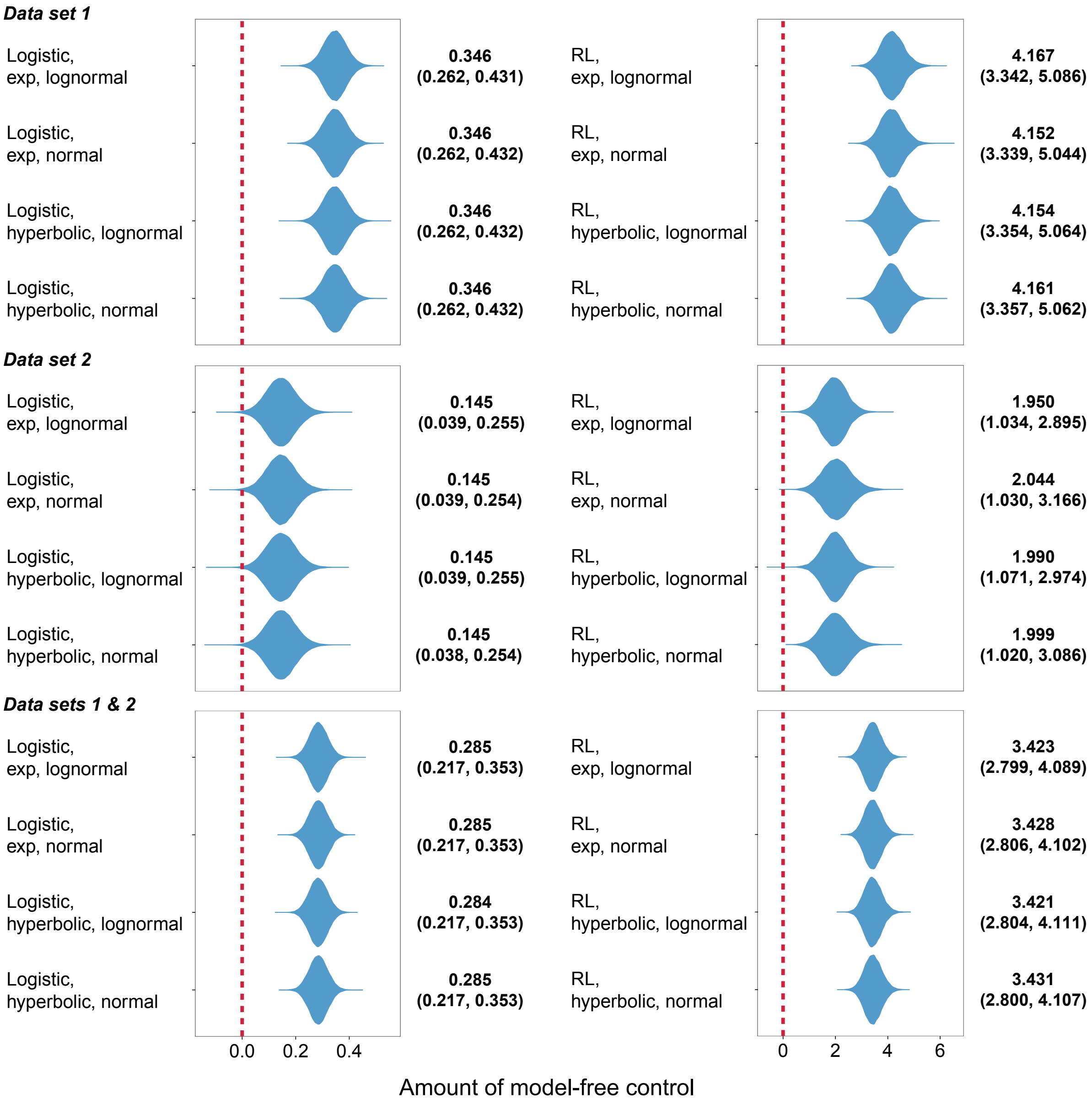


Figure S2: Violin plot of the posterior distribution of the estimated amount of model-free control in the two-step task under each model formulation. Beside each plot is the median value and the 95% credible interval.

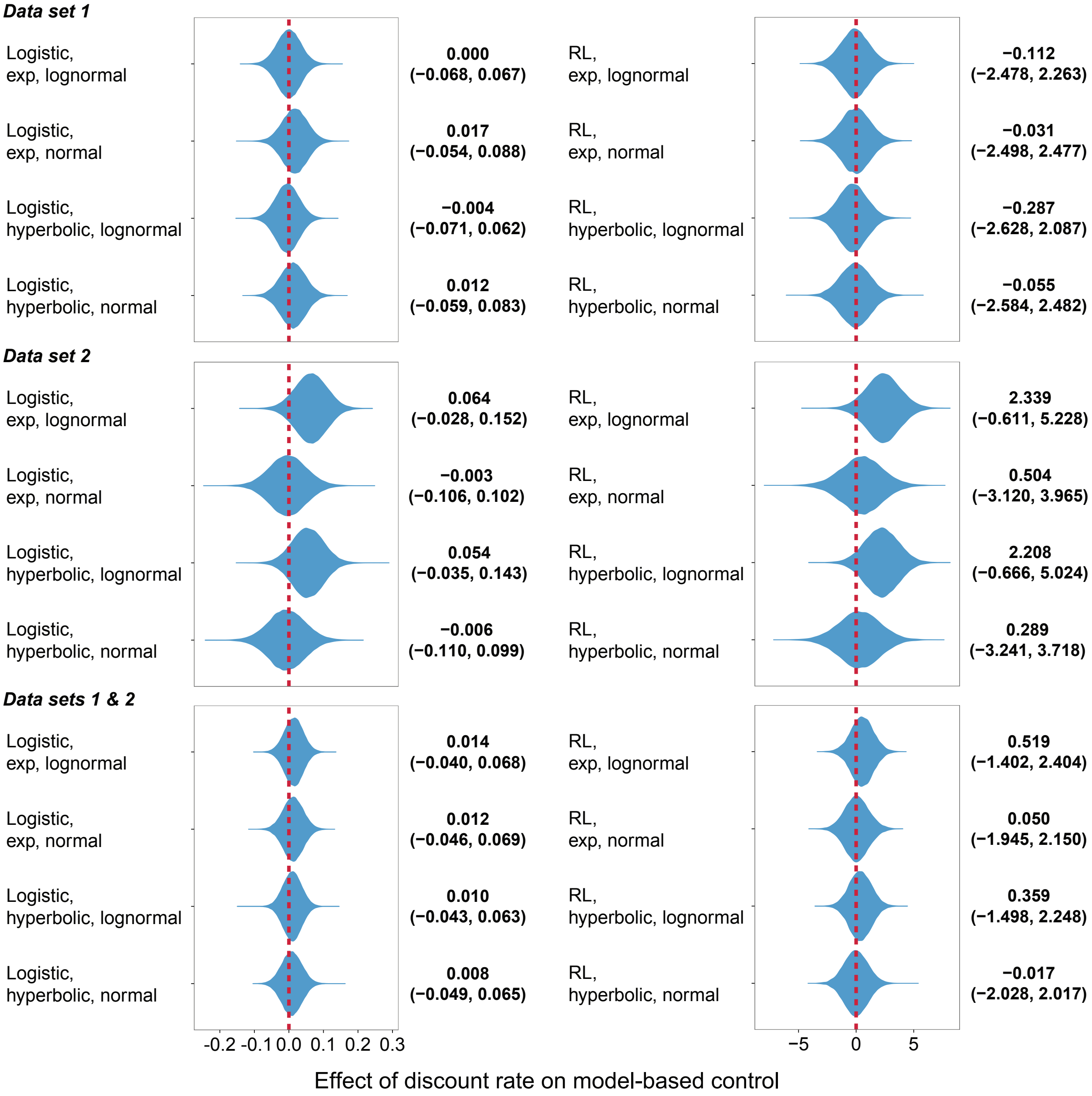


Figure S3: Violin plot of the posterior distribution of the regression coefficient modeling the effect of discount rate in the intertemporal choice task on model-based control in the two-step task under each model formulation. Beside each plot is the median value and the 95% credible interval. This analysis only includes participants whose 95% credible interval for model-based control, model-free control, or control at the second stage includes 0 when fitting the two-step task separately.

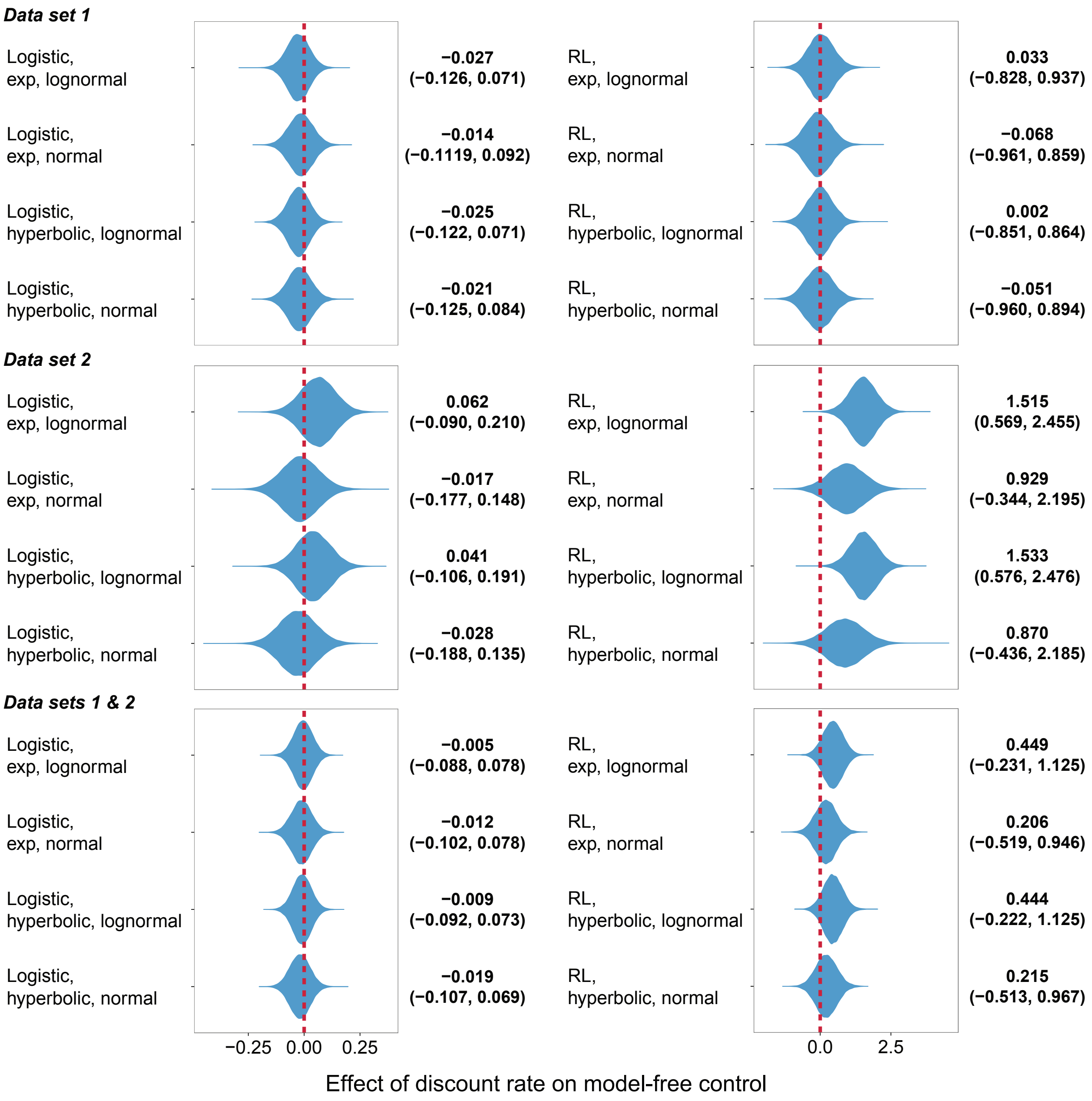


Figure S4: Violin plot of the posterior distribution of the regression coefficient modeling the effect of discount rate in the intertemporal choice task on model-free control in the two-step task under each model formulation. Beside each plot is the median value and the 95% credible interval. This analysis only includes participants whose 95% credible interval for model-based control, model-free control, or control at the second stage includes 0 when fitting the two-step task separately.

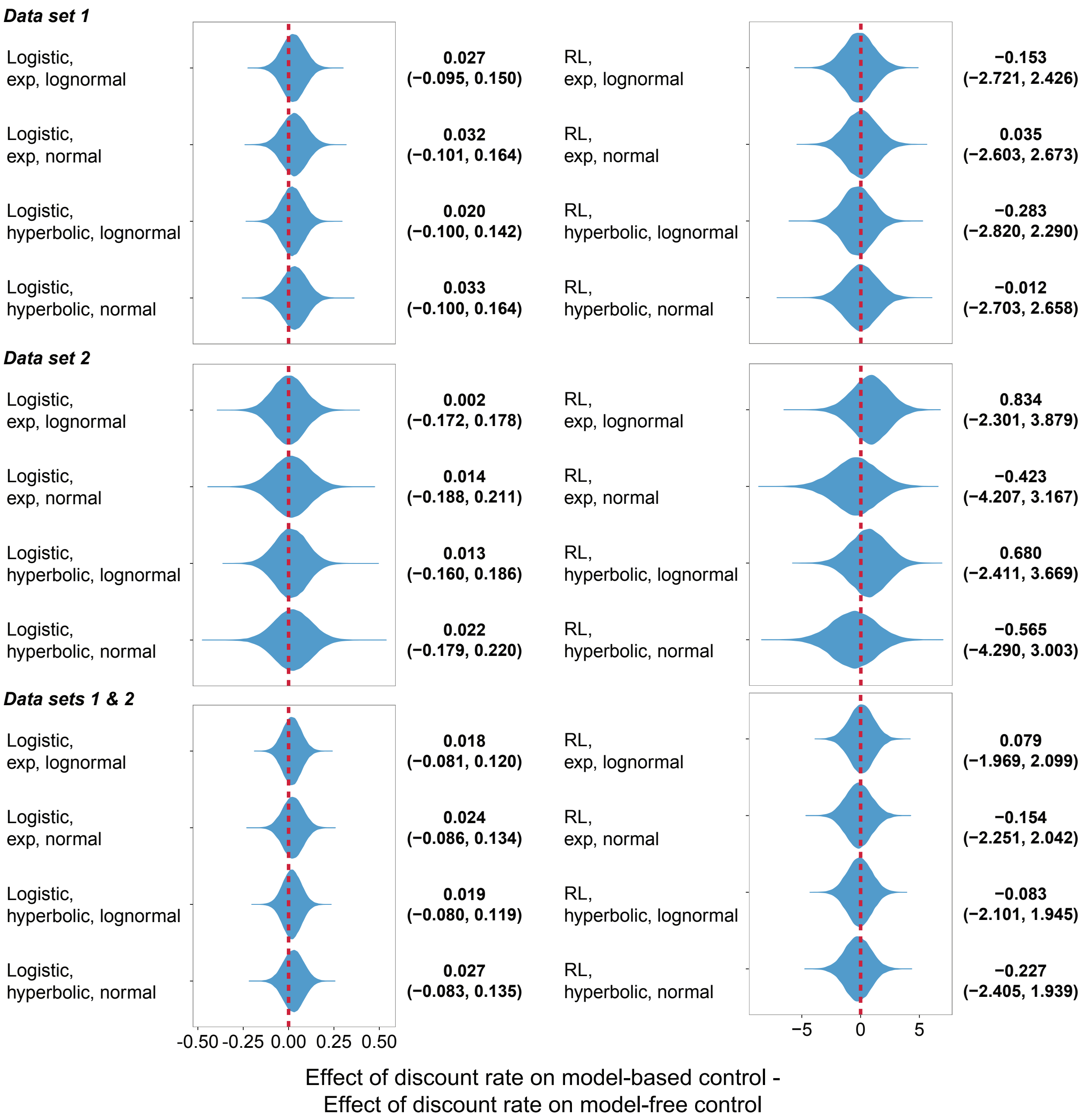


Figure S5: Violin plot of the posterior distribution of the differential effect of discount rate in the intertemporal choice task on model-based control in the two-step task under each model formulation. Beside each plot is the median value and the 95% credible interval. This analysis only includes participants whose 95% credible interval for model-based control, model-free control, or control at the second stage includes 0 when fitting the two-step task separately.
